# Mercury Poisoning at a Home Day Care Center — Hillsborough County, Florida, 2015

**DOI:** 10.15585/mmwr.mm6617a1

**Published:** 2017-05-05

**Authors:** Mackenzie Tewell, Samantha Spoto, Michael Wiese, Alfred Aleguas, Tamas Peredy

**Affiliations:** ^1^Florida Department of Health in Hillsborough County; ^2^Arizona Department of Health Services; ^3^Florida Poison Information Center Tampa.

On November 12, 2015, the Florida Poison Information Center Tampa notified the Florida Department of Health in Hillsborough County of a boy aged 3 years with a urine mercury level of 79 *μ*g/L (normal <10 *μ*g/L). The patient had been admitted to the hospital on October 9, 2015 after a 3–4 week history of anorexia, weight loss, and lethargy. In the hospital, he developed a maculopapular rash, acrodynia (painful, pink discoloration of the hands and feet), tachycardia, hypertension, weakness, sweating, excessive salivation, and altered mental status. Subsequent investigation identified the source of the mercury exposure to be a broken sphygmomanometer (blood pressure monitor) at the home day care center attended by the child.

## Investigation and Results

The patient’s father was interviewed to identify exposures to mercury, such as fish, employment industries or hobbies, or broken mercury-containing items, including thermometers, sphygmomanometers, and fluorescent light bulbs (*1*). No apparent exposures were identified, and on November 13, the family’s rented home was tested by the Florida Department of Environmental Protection, which found no evidence of contamination. On November 19, the child began chelation therapy according to recommendations of the Southeast Pediatric Environmental Health Specialty Unit* and the Florida Poison Information Center Tampa.

Because the patient’s clinical signs and symptoms suggested chronic exposure to mercury vapors, investigators focused on a large family-run home day care center attended by the child (*2*). The day care center owner was interviewed to identify possible exposure sources and was asked about illnesses among other children and staff members (*1*). No similar illnesses were reported; however, the owner reported purchasing a sphygmomanometer at an antique auction and placing it in the children's play area in early July, with the intention of providing a realistic experience for learning. The device was removed 3 weeks later because the children had pulled off the two attached hoses. The day care center owner was not aware that the sphygmomanometer contained mercury, and no loose mercury was observed.

## Public Health Response

Mercury testing of the day care center was conducted on November 18, and revealed hazardous mercury vapor levels as high as 89 *μ*g/m^3^. The day care center was immediately closed, and the home’s residents were advised to relocate until remediation was completed. On November 19, an Environmental Protection Agency (EPA) response team coordinated with local contractors to complete the home remediation. A small bead of mercury was observed in the sphygmomanometer, which had been stored in the family’s unattached garage after its removal from the home. EPA estimated that approximately 80 g (6 mL) of mercury were originally in the sphygmomanometer. The carpet, carpet pads, and other mercury-contaminated household items were removed; beads of mercury were observed on the floor when the carpets were removed. The floors were cleaned with an Epsom salt wash, followed by heating and ventilation of the home. The remediation was completed within 2 days, and the home was cleared by EPA to reopen when mercury concentrations were 1.63 *μ*g/m^3^, a level consistent with the Agency for Toxic Substances and Disease Registry’s recommended action level for mercury vapors in schools (≤3 *μ*g/m^3^) (*3*). Hillsborough County Child Care Licensing inspected the home on November 25, and the day care center resumed operation on November 30, after the Thanksgiving holiday.

Parents of day care attendees and persons who visited the day care home since July 2015 were informed about the exposure and advised to be screened for heavy metal exposure. To determine whether children might have tracked mercury out of the day care center on their shoes or clothes, parents’ cars were screened for mercury. All cars screened negative; therefore, screening of any of the children’s homes was deemed unnecessary.

A case of day care center–associated mercury exposure was defined as a blood or urine mercury level >10 *μ*g/L in a person exposed at the day care center and a diagnosis of mercury poisoning by a medical professional. Twenty-six potential exposures were identified, excluding parents,^†^ who only entered the facility briefly to drop off and pick up their children. A total of 23 persons were tested, among whom 13 (57%) met the case definition, including 10 day care attendees (*4*); the median age of attendees was 2.6 years (range = 1–4 years). The remaining three cases occurred in residents of the home (median age = 54 years). Testing was recommended, but not completed, for one adult and two children who occasionally visited the home in the evenings. Most persons with elevated mercury levels were asymptomatic or experienced mild symptoms; however, some self-reported and parent-reported symptoms of long-term exposure included anxiety, excessive shyness, irritability, eye irritation, vision changes, hypertension, and excessive sweating. Two additional cases were identified in children who were residents of another state and had visited the home; they received chelation therapy by their primary care providers.

Succimer, an oral chelating agent used to remove lead and heavy metals from the body, was recommended by the Southeast Pediatric Environmental Health Specialty Unit and the Florida Poison Information Center Tampa for seven patients. Although approved for the treatment of lead poisoning, succimer is used off-label for treating mercury toxicity (*5*). All seven patients received a 19-day course of succimer. After treatment, five patients had urine mercury levels that remained above reference ranges. None received additional chelation treatment. As of March 2016, all patients had mercury levels <10 *μ*g/L with no signs or symptoms of mercury toxicity.

## Discussion

Children are at increased risk for exposure to mercury poisoning because they are more likely to play on floors and because their smaller lung capacity facilitates increased breathing of mercury vapors (*2*,*3*). Little is known about long-term effects of exposure to mercury vapors in children; however, the central nervous system is most affected by mercury vapor exposure, and long-term cognitive impairments have been observed (*6*,*7*).

As a result of this investigation, the Florida Department of Health developed an educational health notice about mercury for day care providers highlighting the dangers associated with exposure to mercury from broken medical devices such as thermometers and sphygmomanometers (*8*). The Florida Department of Children and Families, who regulate day care centers, were provided with copies of a mercury fact sheet to include in their direct mail outreach and also emailed the fact sheet to other facilities. When day care settings are identified as possible sites of mercury exposure, every effort should be made to explore and test these sites thoroughly. Failure to educate day care staff members about the types of items that contain mercury could place attendees and staff members at risk. Including education about mercury poisoning in day care licensing and regulatory guidelines could reduce mercury exposure to young children in these settings.

**Summary**

**What is already known about this topic?**

When mercury is spilled indoors it can result in numerous health effects, particularly among young children with developing nervous systems. The relative rarity of mercury toxicity might make attributing clinical signs and symptoms to mercury poisoning difficult. Thus, epidemiologic investigations rely on identification of sources of mercury and exposure locations.

**What is added by this report?**

In November 2015, 13 cases of mercury poisoning were detected among attendees and residents of a home day care center after identification of elevated urine mercury levels in a hospitalized child who attended the day care center. The source of the mercury was an antique sphygmomanometer that was placed in the day care center as an educational toy. The owners were unaware that the device was leaking elemental mercury without appearing to be broken. Exposure continued for nearly 6 months before detection during an epidemiologic investigation.

**What are the implications for public health practice?**

Although the awareness of the dangers of mercury-containing items has increased over time, exposure to mercury can still occur. A home day care center serves as both a home and a business, and hobbies, collections, cultural practices, and occupational exposures of residents in the home might inadvertently expose day care attendees. Education and regulation of mercury containing items among home day care providers could prevent exposures leading to serious health effects.

## Footnotes

* Pediatric Environmental Health Specialty Units are located in 10 U.S. regions to provide education and consultation for health professionals, public health professionals, family members, and others about children's environmental health. Pediatric Environmental Health Specialty Units were formed as part of its ongoing cooperative agreements with the Agency for Toxic Substances and Disease Registry and the Environmental Protection Agency.

† One parent who was pregnant was advised to be tested out of an abundance of caution; she had urine mercury levels below the reference minimum.
